# Are C-reactive protein concentrations affected by smoking status and physical activity levels? A longitudinal study

**DOI:** 10.1371/journal.pone.0293453

**Published:** 2023-11-09

**Authors:** Diego G. D. Christofaro, Raphael M. Ritti-Dias, William R. Tebar, André O. Werneck, Márcio S. Bittencourt, Gabriel G. Cucato, Raul D. Santos

**Affiliations:** 1 Departament of Physical Education, São Paulo State University - UNESP, Presidente Prudente, Brazil; 2 Graduated Program in Rehabilitation Sciences, Universidade Nove de Julho - UNINOVE, São Paulo, Brazil; 3 Centro de Pesquisa Clínica e Epidemiológica, Hospital Universitário, Universidade de São Paulo – USP, Sao Paulo, Brazil; 4 Faculdade de Saúde Pública, Universidade de São Paulo – USP, Sao Paulo, Brazil; 5 Faculdade Israelita de Ciências da Saúde Albert Einstein, Sao Paulo, Brazil; 6 Northumbria University, Newcastle Upon Tyne, United Kingdom; 7 Hospital Israelita Albert Einstein, Sao Paulo, Brazil; 8 Heart Institute (InCor) University of São Paulo Medical School Hospital, Sao Paulo, Brazil; Saga University, JAPAN

## Abstract

**Background and objective:**

To compare high-sensitivity C-reactive protein (hsCRP) levels according to smoking status and physical activity (PA) changes in adults.

**Methods:**

The sample consisted of 6028 participants (4833 men) who underwent a voluntary routine health evaluation at the Preventive Medicine Center at the Hospital Israelita Albert Einstein, Sao Paulo, Brazil, from January 2007 to December 2013. Data were collected at baseline and follow-up (2.7±1.6 years). Plasma hsCRP (in mg/L) was analyzed in both moments. Smoking status was obtained through a self-reported questionnaire, being participants classified as non-smokers, once smokers (report smoking at baseline or follow-up), and persistently smokers (reported smoking at both baseline and follow-up). PA was assessed by questionnaire in both moments, being participants classified as persistently inactive, became inactive, became active, and persistently active. The Rank Analysis of Covariance was used to compare hsCRP follow-up values according to smoking and physical activity status.

**Results:**

Persistently smokers showed significantly higher median values of hsCRP at follow-up (1.3 mg/L, IQR:0.6–2.8) than once smokers (1.1 mg/L, IQR: 0.6–2.4) and non-smokers (1.0 mg/L, IQR: 0.5–2.2), even considering covariates (p<0.001). Persistently actives had lower levels of hsCRP at follow-up when compared to persistently inactive in the three smoking status groups (non-smokers p<0.001, once smokers p = 0.001, and persistently smokers p = 0.037).

**Conclusion:**

Persistently active participants had lower hsCRP values at follow-up than those persistently inactive in all the smoking status groups. Regular practice of PA is an important strategy for facing low-grade inflammation, even among smokers.

## Introduction

Smoking elicits several deleterious health consequences, including low-grade inflammation, which is characterized by an elevated plasma high-sensitivity C-reactive protein (hsCRP) [1]. This is important issue since increased hsCRP levels are associated with elevated cardiovascular risk and higher mortality rates [2].

The short-term strategies for reducing cardiovascular risk among smokers are of most importance, given the challenging nature of achieving smoking cessation in short period. It has been reported that approximately 50% of adult smokers have been dependent on nicotine for several years [3], and 35% of participants in treatment for nicotine dependence quit within the first six months [4]. In this sense, maintaining persistent engagement in physical activities could be a strategy for reducing cardiovascular risk, as it contributes to lower hsCRP levels even among current smokers.

Previous studies have demonstrated that physical activity is inversely associated with hsCRP [5, 6] in non-smoking individuals. However, when considering current smokers, it needs to be clarified whether physical activity could contribute to beneficial effects on hsCRP to the same extent as non-smokers. Moreover, the longitudinal potential impact of physical activity on hsCRP in smokers is unknown.

Therefore, the aim of this longitudinal observational study was to compare hsCRP levels among different smoking status groups based on changes in physical activity. The goal was to observe whether participants who maintained persistent activity over 2 years would exhibit lower hsCRP levels compared to their counterparts in the various smoking status groups.

## Methods

### Study sample

We included all adults aged 18–60 who participated in an employer-sponsored routine health evaluation at the Jardins Unit of Hospital Israelita Albert Einstein (Sao Paulo, Brazil) from January 2007 to December 2013. For the present analysis, we included all individuals who underwent two separate visits within the program. For individuals with multiple return visits, the latest visit (median follow-up of 2.0 years with interquartile range—IQR 1.0–4.0) was used for the follow-up analysis. The. The study protocol was conducted in accordance with the Declaration of Helsinki and was approved by Hospital Israelita Albert Einstein’s ethics committee (protocol number CAAE: 35855520.8.0000.0071). All participants signed an informed consent form agreeing to participate in the study.

To calculate the sample size of the present study, we considered a hsCRP standard deviation of 1.20 for non-smokers and 1.30 for current smokers [7], sample power of 80%, and alpha error of 5%, which generated a minimum sample of 2435 participants. However, due to the study’s longitudinal design and the potential for participant attrition, an additional 50% was added to the initial sample size, resulting in a total minimum sample size of 3653 participants.

**Fig 1:**
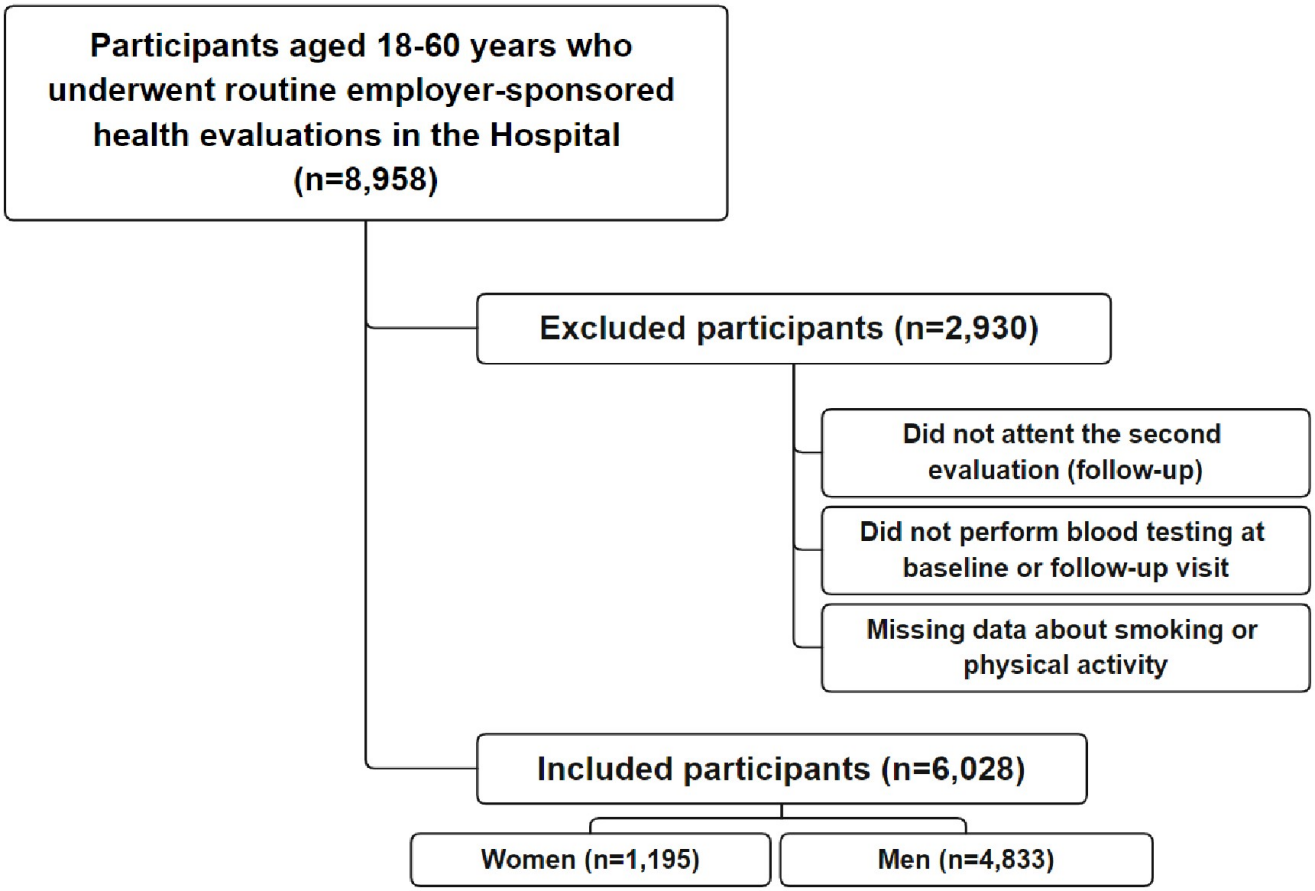
Sampling flowchart.

Initially, 8958 participants started the study, but 2930 were excluded for several reasons: failure to attend the second evaluation, failure to perform a blood test at any time, and no response at any of the smoking or physical activity questionnaire moments. Therefore, the final sample comprised 6028 participants (4833 men and 1195 women). The sampling flowchart is presented in the Fig 1.

### Co-morbidities, risk factors for cardiovascular disease, and medication use

Elevated waist circumference was defined as ≥ 80 cm for women and ≥ 94 cm for men. Body mass index (BMI) was defined as the individual’s weight (kg) divided by the square of their height (m^2^). The difference between BMI from visit one to follow-up was also calculated. Blood pressure was measured three times at the sitting position with an aneroid sphygmomanometer according to the standard method recommended by the American Heart Association [8]. Arterial hypertension was defined by average blood pressure values ≥140/90 mmHg or by previous use of blood pressure lowering medications. Blood samples were collected after an overnight fast. Elevated triglycerides were defined as ≥150 mg/dL, while elevated fasting glucose was defined as ≥100 mg/dL or using lipid or glucose lowering medications, respectively. Low HDL-cholesterol was defined as < 50 mg/dL for women and < 40 mg/dL for men [9]. Subjects were questioned about lipid-lowering medications that can influence hsCRP levels (statins, ezetimibe, and fibrates) at baseline and follow-up [10, 11]. These variables were considered adjustments in the statistical analysis due the strong relationship with hsCRP.

### Smoking status

Smoking habit was obtained in both moments by a face-to-face interview. The following questions were asked of the participants considering their smoking habits:

Have you smoked a cigarette in the last thirty days?

The subjects were classified according to their smoking habits: non-smokers, once smokers (smoker at baseline or follow-up), and persistently smokers (smoker at baseline and no follow-up).

### Physical activity levels

Physical activity status was evaluated at baseline and follow-up through the short form of the International Physical Activity Questionnaire (IPAQ). This instrument was previously validated for the adult population [12]. This questionnaire considers the practice of physical activity in commuting activities, physical activity during leisure, and physical activity, considering weekly frequency and moderate to vigorous intensity. Participants were classified as sufficiently active when the practice of moderate to vigorous intensity physical activity was equal to over 150 minutes per week and inactive when values were less than 150 minutes. Subsequently, four subgroups were created: i) Persistently inactive participants (inactive at both times); ii) Become inactive (active/inactive); iii) Become active (inactive/active) and persistently active (baseline and follow-up).

### Low-grade inflammation

The measurements of hsCRP were performed at baseline and follow-up, where blood plasma samples were collected after an overnight fast and analyzed as part of the routine clinical workflow. Laboratory analyses included the determination of hemoglobin concentrations, a standard lipid panel, and fasting glucose using a Vitros platform automated laboratory system (Johnson & Johnson Clinical Diagnostics, New Brunswick). Low grade inflammation was defined as hsCRP concentrations >3.0mg/L [13] in the absence of infectious and inflammatory diseases. The changes in hsCRP levels were further calculated (2nd visit minus 1st visit values).

### Statistical analysis

The sample characteristics were described in median and IQR for continuous variables and in frequency and 95% confidence interval (CI) for categorical variables. The comparison of hsCRP according to changes in physical activity (persistently inactive; became inactive; became active, and persistently active) was stratified into smoking status (no smoker; once smoker, and persistently smoker) and assessed by Rank Analysis of Covariance (Rank ANCOVA): i. dependent variable (follow-up hsCRP plasma concentration) and covariates (sex, age, follow-up time, changes in abdominal circumference, hypertension status, triglycerides, HDL-cholesterol, glucose and lipid lowering medications) were firstly ranked, ii. Unstandardized residuals of linear regression between dependent variable and covariates were saved; iii. ANOVA with residuals as the dependent variable and physical activity as a factor; iv. posthoc of Bonferroni for identification of differences between groups. A fully adjusted Poisson regression model with a robust variance estimator was used to analyze the association of hsCRP follow-up levels with physical activity in each smoking status group, considering changes in physical activity as a factor and persistently inactive as the reference category. A sensitivity analysis of Poisson regression was performed by stratifying the participants according to follow-up time (1 year or less; 2–3 years, > 3 years). The interaction effect between physical activity and smoking status was analyzed by line graph plotting median hsCRP levels at follow-up according to physical activity categories at the vertical axis and smoking status at the horizontal axis, considering no interaction if lines presented parallel behavior. The interval confidence adopted was 95%, and the statistical significance was 5%. The statistical package used was SPSS 24.0.

## Results

The baseline clinical and laboratory characteristics of sample is presented according to smoking habits–Table 1. The mean age of the sample was 50.2 ±9.0 years, 80.2% men, and the mean BMI was 26.4 ±3.8.kg/m^2^. The prevalence of non-smokers was 72.5% (n = 4369), smokers at one time (once smokers) 19.3% (n = 1162), and persistently smokers 8.2% (n = 497). The prevalence of high hsCRP (>3.0mg/dL) at baseline increased according to smoking status, being 16.5% among non-smokers, 17.9% among once smokers, and 21.9% among persistently smokers (p = 0.003), and decreased according to physical activity, 21.6% among persistently inactive, 19.9% among those who became inactive, 15.0% among those who became active, and 11.5% among the persistently active group (p<0.001). The proportion of persistently inactive participants was 39.8% (n = 2402), those who became active was 20.4% (n = 1229), those who became inactive was 12.1% (729), and the proportion of persistently active participants was 27.7% (n = 1668).

**Table 1:** Baseline clinical and laboratory characteristics of the study sample according to smoking habit (n = 6028).

	Non-smokers (n = 4369)	Once smokers (n = 1162)	Persistently smokers (n = 497)	p-value*
**Variables**	**Median (IQR;25–75)**	**Median (IQR;25–75)**	**Median (IQR;25–75)**	
Age (years)	49.0 (43.0–55.0)	54.0(46.0–60.0)	50.0 (43.0–57.0)	<0.001
BMI (kg/m^2^)	25.8 (23.6–28.2)	26.8 (24.7–29.1)	26.6 (24.3–29.0)	<0.001
WC (cm)	93.0 (85.0–100.0)	97.0 (89.0–103.5)	95.0 (88.0–102.0)	<0.001
hsCRP (mg/L)	1.0 (0.5–2.2)	1.3 (0.6–2.5)	1.3(0.7–3.0)	<0.001
SBP (mmHg)	115.0 (110.0–120.0)	120.0 (110.0–122.0)	120.0 (110.0–120.0)	<0.001
DBP (mmHg)	75.0 (70.0–80.0)	80.0 (70.0–80)	80.0 (70.0–80)	<0.001
Triglycerides (mg/dl)	107 (79.0–153.0)	121.5 (88.0–168.00)	133.0 (92.0–200.5)	<0.001
HDL-C (mg/dl)	48.0 (40.0–57.0)	47.0 (40.0–56.0)	46.0 (38.0–55.0)	<0.001
Glucose (mg/dl)	87.0 (82.0–93.0)	89.0 (84.0–96.0)	88.0 (83.0–95.0)	<0.001
	**Frequency (95%CI)**	**Frequency (95%CI)**	**Frequency (95%CI)**	**p-value** **
**Sex (%)**				
Men	78.3 (77.0–79.5)	85.8 (83.8–87.8)	83.9 (80.7–87.1)	<0.001
Women	21.7 (20.5–23.0)	14.2 (12.2–16.2)	16.1 (12.9–19.3)	
**CRF (%)**				
High hsCRP	16.5 (15.3–17.4)	17.9 (15.7–20.1)	21.9 (18.2–25.7)	0.003
Hypertension	14.3 (13.3–15.4)	16.3 (14.2–18.8)	14.2 (11.2–17.3)	0.407
High Triglycerides	11.3 (10.4–12.2)	15.6 (13.5–17.6)	24.9 (21.2–28.7)	<0.001
Low HDL-cholesterol	22.3 (21.1–23.6)	24.8 (22.3–27.3)	29.4 (25.3–33.4)	<0.001
High fasting glucose	8.9 (8.1–9.8)	16.8 (14.7–19.0)	9.9 (7.2–12.4)	<0.001
Lipid lowering medications	14.8 (13.7–15.8)	23.9 (21.4–26.3)	17.1 (13.8–20.4)	<0.001
**Physical activity (%)**				
Persistently inactive	39.6 (38.1–41.0)	38.5 (35.7–41.3)	45.7 (41.3–50.1)	0.127
Became Inactive	11.7 (10.8–12.7)	14.2 (12.2–16.2)	10.5 (7.8–13.2)	
Became active	20.8 (19.6–22.0)	18.8 (16.6–21.1)	20.1 (16.6–23.7)	
Persistently active	27.9 (26.6–29.2)	28.5 (25.9–31.1)	23.7 (20.0–27.5)	

*Kruskal Wallis test;

**Chi-square test for linear-by-linear association;

IQR: Interquartile range; WC: waist circumference; CRF: Cardiovascular risk factors; High hsCRP: ≥3.0mg/L; Hypertension: blood pressure values ≥140/90mmHg or use of blood pressure lowering medications; High triglycerides: ≥150mg/dL; High fasting glucose: ≥100mg/dL; Low HDL-cholesterol: <50mg/L for women and <40mg/L for men.

**Fig 2:**
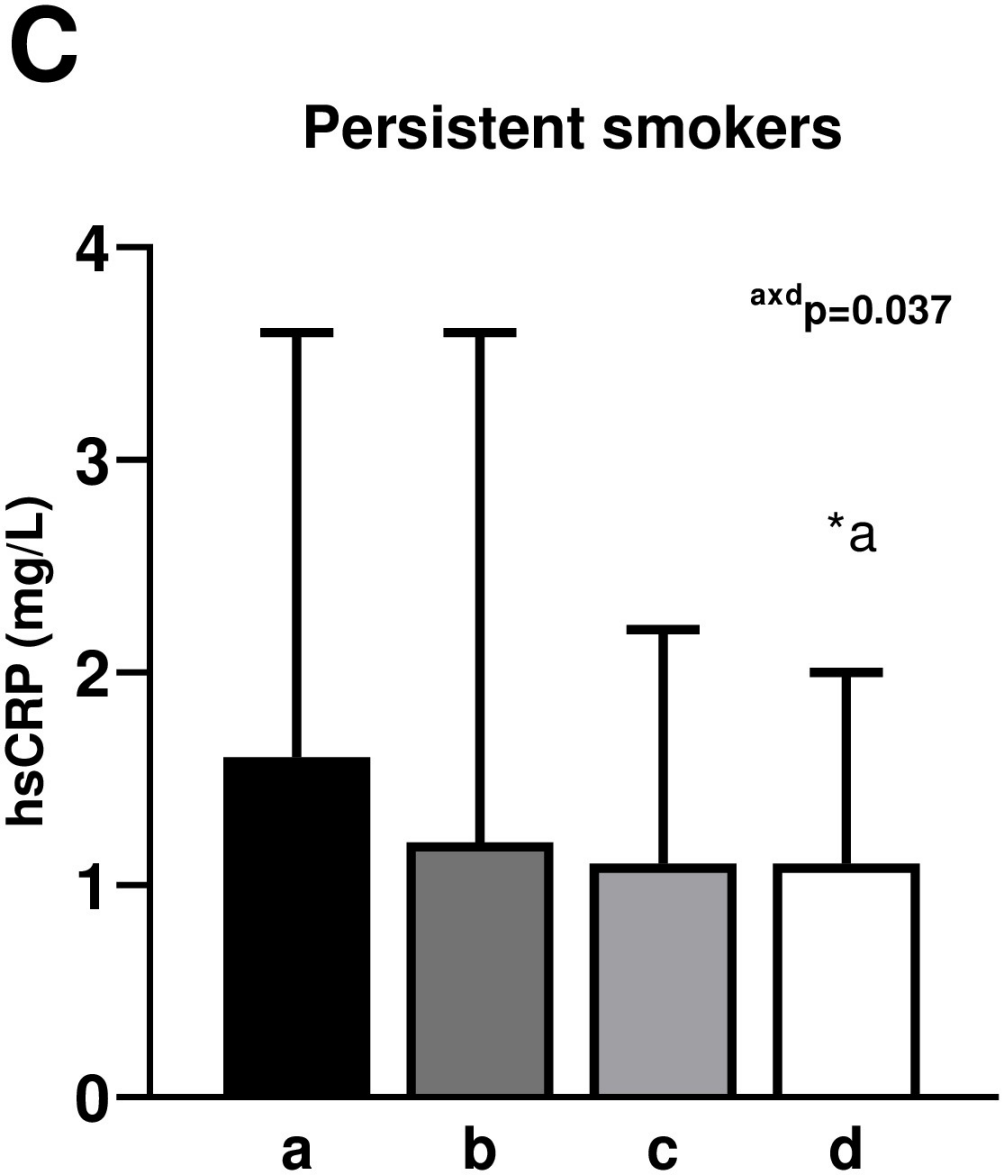
Median and interquartile range values of high-sensitivity C-reactive protein at follow-up according to smoking status and changes in physical activity (n = 6028). Rank Analysis of Covariance adjusted by sex, age, time of follow-up, changes in waist circumference, hypertension, plasma triglycerides, HDL-cholesterol, glucose, and lipid lowering medications; a = Persistently inactive; b = Became inactive; c = Became active; d = Persistently active.

The Fig 2 shows the comparison of hsCRP values at follow-up according to physical activity engagement among non-smokers, once smokers, and persistently smokers. Among non-smokers, participants who were persistently active had lower values of hsCRP at follow-up than all the other groups (persistently inactive [p<0.001], became inactive [p = 0.019], and became active [p = 0.034] groups). Regarding once smokers, persistently active participants had lower hsCRP values at follow-up than those who were persistently inactive (p = 0.001) and those who became inactive (p = 0.005). In the persistently smokers group, persistently physically active participants had lower hsCRP values at follow-up than those persistently inactive (p = 0.037). All these differences were observed after adjustment for sex, age, follow-up time, changes in abdominal circumference, hypertension status, triglycerides, HDL-cholesterol, glucose, and lipid lowering medications.

The median changes in hsCRP levels between the two visits was zero (P25 = -0.6; P75 = 0.5). No difference in values of hsCRP change (2nd visit minus 1st visit values) was observed according to smoking status (p>0.05). When stratified to smoking status, no difference in values of hsCRP change was observed according to physical activity in persistent smokers (p = 0.796). Although statistically significant, extremely slight variations in hsCRP over time were observed across physical activity categories among non-smokers and once smokers, ranging from 0.1 for persistently inactive to -0.1 for persistently active in both smoking status groups (p<0.05).

**Fig 3:**
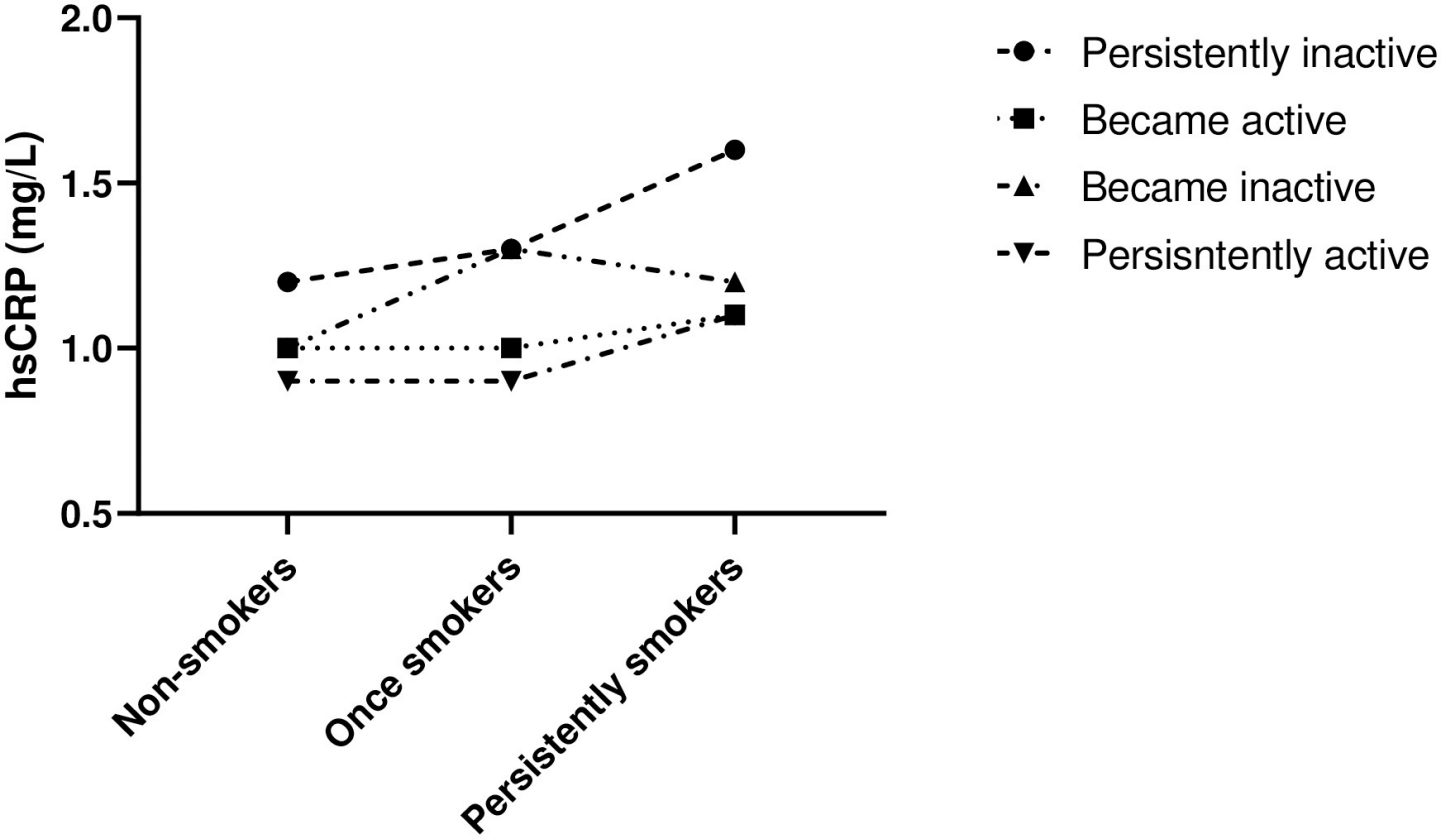
Interaction plot between physical activity and smoking status over high-sensitivity C-reactive protein follow-up values.

The interaction plot demonstrated that lines of hsCRP median values at follow-up were not parallel and a steeper line among persistently inactive participants across the smoking status categories, showing a potential interaction effect between physical activity and smoking status over hsCRP level–Fig 3.

The multivariable Poisson regression analysis showed significantly lower hsCRP levels (β = -0.676 [95% CI: -1.14; -0.21], p = 0.004) in the persistently active group of persistent smokers when compared to those who were persistently inactive. Persistently active participants from the non-smoker and once smoker groups showed no association with lower hsCRP levels compared to persistently inactive participants from their respective groups. The sensitivity analysis considering participants with 1 year or less follow-up and between 2–3 years of follow-up showed no association between physical activity and hsCRP levels in the three smoking status strata. However, when considering the participants with a longer follow-up (>3 years), being persistently active was associated with lower hsCRP levels among persistently smokers (β = -0.71 [-1.33; -0.08] p = 0.028). Another important finding among participants with longer follow-up was that becoming physically active from the first to second visit was associated with lower hsCRP values among once smokers (β = -1.52 [-2.61; -0.43] p = 0.006) and persistently smokers (β = -1.04 [-1.97; -0.10] p = 0.029).

## Discussion

The main findings of this study were that participants who became active or were persistently active had lower hsCRP plasma levels compared to sedentary ones, regardless of smoking status. In addition, once smoker or persistently smoker participants had a poor metabolic profile at baseline and follow-up.

Smoking have been associated with inflammation of the respiratory system, especially in the bronchi, which could lead to the increase of cytokines like interleukin-6 and tumor necrosis factor (TNFα) that modulate the production of hsCRP by the liver [14, 15]. Kianoush et al. [1], in a study with more than 11,000 adults, observed that smoking was associated with higher plasma hsCRP. Azar and Richard, in a study evaluating salivary C-reactive protein levels in the context of tobacco smoke exposure, found that hsCRP values were higher in active smokers when compared to passive or nonsmoking smokers [16].

The main recommendation to improve the health of smokers is the smoking cessation cigarette, however, its impact on low-grade systemic inflammation is controversial. Gallus et al. [17], using two screening studies conducted in Italy in 2000–2010, found that smoking cessation was positively associated with lower plasma hsCRP levels, but this benefit was not verified in the short term. Aldaham et al. [18] did not observe significant differences in the levels of hsCRP in current and former heavy smokers.

Another important recommended lifestyle behavior to control low-grade inflammation is physical activity practice. A previous study observed an inverse association between higher levels of vigorous physical activity and hsCRP in US adults [19]. In addition, Pitanga et al. [20], evaluating 822 Brazilian adults, observed that in men, physical activity was considered a protective factor against high hsCRP levels, even in smokers. However, both studies were cross-sectional design, which prevents the analysis of cause and effect. In our longitudinal study, we observed that subjects who became active and were not smokers or who were smokers in a single moment of the follow-up had lower hsCRP levels than sedentary ones. Moreover, when considering the persistently active individuals during follow-up, lower values of hsCRP were observed regardless of the smoking status after adjustment for all potential confounders. Based on the findings, it appears that regularly engaging in physical activity can lead to reduced levels of hsCRP, thus highlighting the significance of promoting exercise to combat low-grade inflammation.

This study must be read within the context of its design. First this is an observational study, not a randomized clinical trial, and therefore subject to residual confounding despite multivariate analysis. Secondly, the sample of the present study consisted solely of workers who participated in routine health evaluations at the study site. Consequently, the sample lacked representativeness and heterogeneity, limiting the generalizability of our findings. Third, we used a subjective measurement of physical activity levels, making it challenging to assess the intensity of the physical activities performed. This aspect is significant to consider since self-reporting the intensity and frequency of physical activity may be susceptible to memory bias [20]. However, it is essential to note that the IPAQ is widely accepted for epidemiological studies and provides a reasonable quantification of physical activity levels [21, 22]. Another limitation is that we only used data from the first and last visit, and it was not possible to identify fluctuations in physical activity and smoking between them. Finally, only smoking habits were evaluated, and the lack of data on the number of cigarettes smoked per day is a limitation to be considered. However, as positive aspects we can quote the robust sample size, its prospective design and medium-term follow-up.

## Conclusion

In summary, persistently active participants had lower hsCRP values, regardless of smoking status and after adjustment covariates. Considering the prognostic role of low-grade inflammation on cardiovascular disease, this study reinforces the role of physical activity in mitigating low-grade inflammation in smokers.
